# *HFE* mutations in patients with iron overload in Santa Catarina: a cross-sectional study

**DOI:** 10.1590/1516-3180.2023.0359.R1.07032025

**Published:** 2025-07-04

**Authors:** Cristiane Manfé Pagliosa, Vivian Karla Brognoli Franco, Thiago Sousa Matias, Bruno Vieira Dias, Andrea Thives de Carvalho Hoepers

**Affiliations:** I Departamento de Nutrição, Universidade do Oeste de Santa Catarina (Unoesc), Joaçaba (SC), Brazil; Clinical Nutritionist and Consultant, Department of Nutrition and Food Sciences at Nectalimentos, Joaçaba (SC), Brazil.; II Centro de Hemoterapia, Hospital Universitário Professor Polydoro Ernani de São Thiago, Universidade Federal de Santa Catarina (UFSC), Florianópolis (SC), Brazil.; III Departamento de Educação Física, Escola de Esportes, Universidade Federal de Santa Catarina (UFSC), Florianópolis (SC), Brazil.; IV Centro de Hematologia e Hemoterapia de Santa Catarina, Florianópolis (SC), Brazil.; V Centro de Hemoterapia, Hospital Universitário Professor Polydoro Ernani de São Thiago, Universidade Federal de Santa Catarina (UFSC), Florianópolis (SC), Brazil.

**Keywords:** Iron overload, HFE protein, human, Mutation, Hereditary hemochromatosis, *C282Y* mutation, *H63D* mutation, *S65C* mutation, *HFE* genotype frequency

## Abstract

**BACKGROUND:**

Investigating the frequency and characteristics of iron overload cases with *HFE* gene mutation is crucial, given the population-level risks associated with excessive iron.

**OBJECTIVE:**

To determine the frequency of *HFE* mutations in patients with iron overload in Santa Catarina, Brazil.

**DESIGN AND SETTING:**

A cross-sectional study of patients with iron overload at the Ambulatory Department of the Centro de Hematologia e Hemoterapia de Santa Catarina (Hemorrede-HEMOSC) in Santa Catarina.

**METHODS:**

***HFE*** genotype frequencies were determined, and a division were made between carriers of ***HFE*** -C282Y/C282Y mutations and carriers of other *HFE*-non-C282Y/C282Y mutations, according to each region of Santa Catarina. Binary logistic regression was used for association between sex and age with genetic mutation trait.

**RESULTS:**

Among the 1,022 patients, 10.4% had secondary hemochromatosis, and 89.6% were evaluated for iron overload due to hereditary hemochromatosis (HH). Of these, 367 underwent genetic testing, which revealed *HFE* mutations in 77.3%. Most patients with *HFE* mutations had non-C282Y/C282Y-hemochromatosis, especially H63D/WT (> 39%), regardless of the Santa Catarina region. The frequency of C282Y/C282Y was higher in the West (20.9%) and North (28.3%) regions. Adjusted association analysis showed that men have an increased chance of hemochromatosis when involving 'non-C282Y/C282Y' mutations (OR: 2.77; 95% CI: 1.60–6.608).

**CONCLUSIONS:**

The data show the magnitude and characteristics of iron overload cases with *HFE* mutations in Santa Catarina. As most patients referred for treatment have H63D mutation, we suggest further studies to assess whether other factors, including dietary habits and mandatory iron fortification policies, contribute to iron overload or HH manifestation.

## INTRODUCTION

 Hereditary hemochromatosis (HH) is an autosomal recessive disease, characterized by an excessive increase in absorption of dietary iron due to alterations in the hepcidin hormone expression, which reduces its concentration and consequently increases the amount of iron accumulation in the human body.^1,2^ Under normal physiological conditions, hepcidin is a negative iron metabolism regulator, i.e., its physical expression is reduced only when there is an increase in the need for iron in the human body.^1,3-5^ Since the human body does not have specific mechanisms to regulate iron excretion, excess iron can be absorbed and cause toxicity. This progressive accumulation of iron leads to the formation of free radicals causing oxidative damage in organs and tissues, especially in the liver, heart and pancreas.^5 ,6^ HH related to the *HFE* gene, defined as type 1, represents more than 90% of the known cases of this disease and appears as mutant alleles C282Y, H63D, and S65C. Penetrance into HH of other *HFE* genotypes could also occur, but would depend on greater exposure to non-genetic factors.^1,4,8^ The homozygous C282Y mutation (C282Y/C282Y) is the most precociuous and most severe iron overload-related subtype among *HFE* mutations.^1,4,5,7^

 Diagnosis of HH is based on the detection of iron overload with elevated serum ferritin level and elevated transferrin saturation level in conjunction with genetic mutations. Early diagnosis and prompt treatment can prevent serious clinical manifestations such as heart disease, arthritis, liver disease, and diabetes.^1,9,10^

 Regular therapeutic phlebotomy (therapeutic bloodletting) is the most effective treatment for HH, which aims to move the accumulated iron in tissues into the blood by inducing a negative balance in serum iron.^2 ,7^ Less exposure to environmental risk factors can attenuate phenotypic expression and facilitate treatment by allowing longer intervals between phlebotomies. Examples of environmental factors include habitual consumption of iron-enriched foods, excessive vitamin C intake, and excessive alcohol consumption.^1,5,8,9,11-14^

 According to data on populations of Caucasian origin, the estimated prevalence of HH type 1 is one case for every 200 individuals.^15 ,16^ Studies on HH prevalence in Brazil are scarce; however, some studies suggest that, in certain regions of the country, the allele frequencies of *HFE* mutations can be comparable to those in countries where HH is considered a public health problem.^17-19^ This is due to Brazil’s genetic heterogeneity, where *HFE* mutations and the phenotypic HH expression could present variations and different frequencies, depending on the colonizing ethnic groups of various regions and the influence of environmental factors.^7,8,12,16,20 -23^

 As data on HH in Santa Catarina is lacking, and to describe the prevalence of type 1 HH cases in a Brazilian population of predominantly European descent, we investigated the frequency of *HFE* mutations in patients from Santa Catarina assisted by the Sistema Único de Saúde (Unified Health System, Brazil’s public healthcare system) through the Centro de Hematologia e Hemoterapia de Santa Catarina (Hemorrede-HEMOSC, the Hematology and Hemotherapy Center of Santa Catarina). 

## METHODS

 A cross-sectional study was conducted using secondary data to describe the frequency of *HFE* mutations in patients with iron overload in Santa Catarina who were referred for investigation and treatment at Hemorrede-HEMOSC. The study was conducted using data from January 1 to December 31, 2016. Cases were selected from medical records by searching for the International Classification of Diseases – 10 (ICD-10) codes E83.1 (iron metabolism diseases) and T4.4 (intoxication by iron and its compounds). For the sampling procedure, all medical records of patients assisted at Hemorrede due to iron overload (ICD-10 and ICD-T4.4) were analyzed. Analyses were conducted between December 2017 and December 2018. 

 To describe the frequency of *HFE* mutations, the exclusion criteria were absence of genetic testing for HH, medical records of patients diagnosed with *HFE*-HH without indicating the mutation type, and patients diagnosed with secondary hemochromatosis (hemoglobinopathies, sideroblastic anemia, polycythemia vera, hereditary spherocytosis, porphyria cutanea tarda, viral hepatitis, or alcoholic liver disease). 

 The data were grouped according to the state’s regions, namely Greater Florianópolis, Itajaí River Valley, North, South, West, and the Serrano Highland.^24^ The mutations frequency observed for the Serrano Highland region is not shown, as no genetic testing data are available for that region. All regions of the state have populations predominantly of European descent, mainly Italians, Germans, and Portuguese; thus, they carry a higher risk of having *HFE* mutations.^25,26^ Information was obtained regarding age, sex, skin color, genotyping test results, as well as the frequency and distribution of *HFE* mutations. Skin color was self-declared by the public healthcare user, within the standards used by the Instituto Brasileiro de Geografia e Estatística (IBGE, the Brazilian Institute of Geography and Statistics). In the public healthcare information systems, these are categorized as white, black, yellow, brown, dark-skinned, or indigenous.^27^ Genotyping for *HFE* mutations is performed by polymerase chain reaction analysis^28^ in laboratories outsourced to HEMOSC. The frequency and distribution of *HFE* gene mutations in patients from Santa Catarina were noted for each region of the state. The age variable was categorized between patients aged ≤ 40 years versus > 40 years, which is the cutoff point referring to the age group for most type 1 HH diagnoses.^2^ For the skin color variable, Santa Catarina’s low prevalence of black, yellow, brown, dark-skinned, and indigenous population was not considered; therefore, only two categories were established, namely White and Non-white.^29^ For the *HFE* genotype frequency analysis, genotypes were divided into six categories, namely, C282Y/C282Y mutation carriers (homozygous C282Y) and carriers of other nonC282Y/C282Y *HFE* genotypes; C282Y/WT (C282Y heterozygous); C282Y/H63D (compound heterozygote C282Y/H63D); H63D/H63D (homozygous H63D); H63D/WT (heterozygous H63D); and all the S65C types as one single group (S65C-grouped). The S65C category included both homozygous and heterozygous patients with this mutation (S65C/S65C, S65C/WT, and C282Y/S65C). Wild-type (WT) was used to signal a non-mutant allele. 

 The collected data were recorded and organized into electronic spreadsheets (Excel), and data files were generated. After the files were checked, they were transferred to statistical analysis software. The data were verified through consensus between two researchers not involved in initial data collection and by reviewing the data the researcher responsible for statistical analysis. The characterization data were described using absolute and relative frequencies, and genotype frequencies (95% CI) were also presented. Binary logistic regression was used to determine the association between sex and age (adjusted analysis) for carriers of the genetic traits (C282Y/C282Y mutation versus other non-C282Y/C282Y *HFE* genotypes). The cut-off for significance was set at 5%, and the data were analyzed using STATA 15 software (Stata Inc., College Station, TX, USA). The research project was approved by the Ethics Committee of the Federal University of Santa Catarina (UFSC) (CAAE: 64252017.2.0000.0121) and the Ethics Committee of the Center for Hematology and Hemotherapy of Santa Catarina (HEMOSC) (CAAE: 64252017.2.3001.0110). This study followed the ethical principles established in Resolutions No. 466/2012 and No. 580/2018 of the National Health Council in Brazil. 

## RESULTS

 During the study period, 1,022 outpatients were assessed for iron overload at Hemorrede-HEMOSC. Of these, 206 (10.37%) had secondary hemochromatosis. The remaining 806 patients (89.63%) were assessed for HH. Of these 806 patients, 367 (35.2%) underwent genetic testing to identify HH, of whom 77.3% tested positive for *HFE* mutations, as shown in Table 1. Of the total number of patients who underwent genetic testing for *HFE* mutations, 89.6% were aged over 40 years, 91.0% were male, and all but one had white skin. 

**Table 1:** Distribution of patients according to age, sex, skin color and presence of genetic alteration in the *HFE* gene in Santa Catarina, Brazil, 2016

**Variable**		**N**	**%**
**Age (years)**	≤ 40	38	10.4
	> 40	329	89.6
	**Total**	**367**	**100**
**Sex**	Male	334	91
	Female	33	9
	**Total**	**367**	**100**
**Skin color**	White	366	96.3
	Non-White**	1	3.7
	**Total**	**367**	**100**
***HFE* gene mutation**	Yes	282	77.3
	Not	83	22.7
	**Total**	365***	**100**

* N: number of patients;

** Brown;

*** Two subjects excluded from the sample (patients diagnosed with ***HFE***-HH, but without indicating the mutation type).

**Figure 1:**
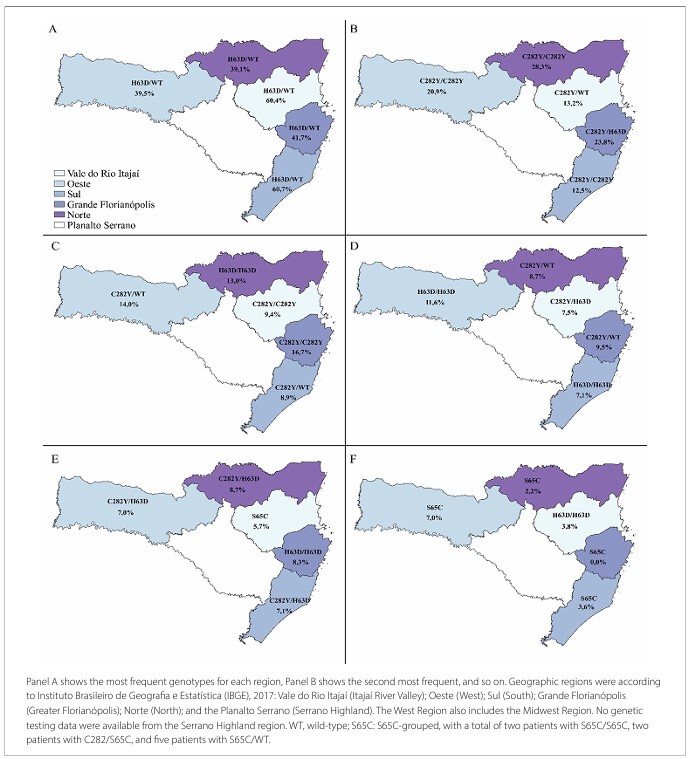
Frequency of *HFE* genotypes in patients, according to each region of Santa Catarina, 2016.

*HFE* genotype frequencies in patients undergoing treatment showed the relevance of the number of iron overload cases in *HFE* mutations in Santa Catarina. Table 2 shows which mutations are involved in these cases, while Figure 1 shows the distribution thereof among the regions. The percentage of patients with homozygous C282Y/C282Y in Santa Catarina was 17%, and the most frequent mutation among patients in this state was heterozygous H63D (48.2%), which was repeated in all regions of the state. The compound heterozygous mutation C282Y/H63D was the third most frequent mutation in the state (12.4%) and the highest in the Greater Florianopolis region (23.8%). 

**Table 2:** Frequency of *HFE* genotypes in patients from Santa Catarina, Brazil, 2016

**HFE genotype**	**N**	**%**	**95% CI**
C282Y/C282Y	48	17	13–21.9
C282Y/WT	30	10.6	7.5–14.8
H63D/H63D	24	8.5	5.7–12.4
H63D/WT	136	48.2	42.4–54.1
C282Y/H63D	35	12.4	9–16.8
S65C-grouped**	9	3.2	1.7–6
**Total**	**282**	**100**	

* N: number of patients in each group; WT: wild type;

** All the S65C types as one single group (two patients S65C/S65C, two patients S65C/C282Y e five patients S65C/WT).

 The non-C282Y/C282Y *HFE* mutation was detected in 83% of the patients and the adjusted analysis association between the sex and age of genetic trait carriers (C282Y/C282Y and other nonC282Y/C282Y *HFE* genotypes) showed that men, have higher chances of having hemochromatosis with non-C282Y/C282Y mutations (OR: 2.77; 95% CI: 1.60–6.608) compared to women. 

## DISCUSSION

 In the present study, cases of iron overload were found predominately in male, white-skinned patients over 40 years of age with mutations in the *HFE* gene, which agrees with reports on HH type 1.^1,2,8^ Although clinical manifestations differ between patients, they tend to be related to the amount of iron accumulated in the body, and they generally begin after 40 years of age, which is the age after which most people are diagnosed with HH.^2,9^ Since 22.7% of patients did not have an *HFE* mutation, some other genetic mutation for HH may be participating, such as genes encoding other proteins involved in iron metabolism, including hemojuvelin (*HJV*), hepcidin (*HAMP*), transferrin receptor (*TfR2*), or ferroportin (*SLC40A1*).^1,5,10^ This proportion of non-*HFE* HH cases is similar to the value of 24% noted by Cançado et al.^22^ in a study involving fifty patients with iron overload being treated in São Paulo. 

 The low S65C mutation frequency in patients from Santa Catarina is similar to the reported *HFE*-HH cases.^21,30,31^ This low frequency is also found in healthy individuals without phenotypic disease expression.^18,32-34^

 The C282Y/C282Y mutation was more common in patients in Santa Catarina than those in Rio Grande do Norte (2.67%) and Espírito Santo (5%), and was similar to that described for São Paulo (21.6%) ^21,30,31^ ; furthermore, it is also similar to a value described in a study in the United States.^35^ Of note, Gallego et al.^35^ investigated 222 men over 59 years of age with the previously known mutation (*HFE* C282Y/C282Y or C282Y/H63D), of whom 24.4% had a clinical diagnosis compatible with HH-*HFE* C282Y/C282Y and 3.4% whose clinical diagnoses were compatible with C282Y/H63D mutation-related HH-*HFE*. 

 When considering the frequency among regions of Santa Catarina, the C282Y/C282Y mutation was least common in Vale do Itajaí and South (Figure 1), with values close to those (10%) reported in another study involving only patients from the South region of Santa Catarina.^36^ The present study shows the heterogeneity of *HFE* mutations, being similar to that observed in southern European countries, such as Italy.^37^

 The highest number of C282Y/H63D patients, compared to those with C282Y/C282Y within Santa Catarina, was noted in the Greater Florianopolis region, which is also where most patients had been screened for *HFE* mutations. In Espírito Santo, a notable association was found between compound heterozygous cases with the H63D mutation and HH when compared to people without phenotypic expression.^31^ In another study conducted in Spain, which has a Mediterranean population, researchers noted greater phenotypic expression for the compound heterozygote mutation C282Y/H63D than other *HFE* mutations,^12^ as seen in patients in the Greater Florianópolis region. The frequency of the C282Y/H63D mutation found (12.3%) was similar to that noted for patients in São Paulo (14–15%)^22,31^ and higher than those in the Northeast Region of Brazil (5.02%),^30^ as well as in Italian (5.3%) and Swedish (7.1%) patients.^37,38^

 The heterozygous H63D mutation showed a similar frequency to that noted in a study conducted in the South region of Santa Catarina, which profiled patients undergoing phlebotomy and being treated at a private hematology clinic, where 44% of patients with *HFE* mutations had heterozygous H63D.^36^ As most patients referred for treatment at HEMOSC have the H63D mutation, regardless of region (Figure 1), additional factors could be contributing to iron overload and HH manifestation in this population.^10^

 Bell et al.^11^ conducted a study in Norway that showed that excessive consumption of iron for at least five years was significantly associated with the clinical hemochromatosis expression in patients with nonC282Y/C282Y *HFE* mutations. Since 2002, the Brazilian population has regularly eaten mandatorily iron-fortified foods (wheat flour, corn flour, and preparations that contain either or both).^39,40^ This may also have contributed to iron overload in carriers of non-C282Y/C282Y *HFE* mutations.^11-13,41-46^ Guidelines advise that patients with HH should avoid daily consumption of iron-fortified foods,^2,8,9^ highlighting the importance of offering such people foods without iron fortification. 

 The present study shows that male sex can be a significant predictor of iron overload development in non-C282Y/C282Y*HFE*-HH cases, suggesting that further studies should assess whether men suffer greater environmental exposure to factors such as excessive iron-rich food consumption (e.g., meat or iron-fortified foods), alcohol, or vitamin C compared to women with the same condition. In addition, it is still unknown whether female physiological conditions could mitigate the effect of exposure to these environmental factors. Further, if this is indeed the case, it remains unknown whether there would be greater relevance for non-C282Y/C282Y *HFE* mutations, since women lose iron during menstruation and require more iron during pregnancy, as well as being affected by the antioxidant effects of estrogen and the naturally higher hepcidin concentrations in women when they are overweight.^46^

 The present study has some limitations. First, the data are from a convenience sample, where patients with iron overload were referred to HEMOSC, although this is the main treatment center for these cases. Second, we lacked data for one of six regions of Santa Catarina. Third, environmental factors, comorbidities, and biochemical data were not investigated; thus, these were not considered in the analytical association model. Next, we could not make many comparisons with other results, since studies in this field are limited in Brazil. Finally, only laboratory tests for *HFE* were used, since they are easily accessible. Laboratories that perform genetic testing for mutations other than those of *HFE* are scarce,^10^ thereby limiting investigations to determine whether mutations in other iron homeostasis-related genes could also be involved. 

 However, the present study describes *HFE* mutation frequency, which is the main HH-related gene, in patients with iron overload treated at a public healthcare center specializing in hematology and hemotherapy in Santa Catarina. Moreover, it can help map and plan public interventions aimed at patients with hemochromatosis and those in at-risk groups, including those with non-C282Y/C282Y mutations. 

 Among such interventions, education is particularly relevant. This could include developing courses and other educational materials to raise awareness about excess iron, its possible causes, and the associated health risks. It could also foster understanding of the importance of early diagnosis by quantifying serum ferritin and transferrin saturation indices in routine examinations among clinical managers. Individuals with laboratory findings suggestive of hemochromatosis or a family history of the condition would then undergo investigation for HH-related mutations. These actions, supported by case mapping, could be expanded to all states in Brazil, strengthening the involvement of different segments of society, including public and private organizations, health professionals, and teaching, extension, and research institutions. Furthermore, recognizing this as a public health issue, it is important to encourage the establishment of associations for patients with HH to seek improvements and share responsibilities for addressing this condition. Another opportunity lies in promoting better coordination between federal public bodies and industries, particularly the flour industry, to facilitate the production and accessibility of common foods without iron supplementation, which would be a crucial measure to mitigate excess iron levels in individuals with this condition. 

## CONCLUSION

 The data obtained in this study showed that most patients with *HFE* mutations have non-C282Y/C282Y hemochromatosis regardless of their region in Santa Catarina, whereas patients with the homozygous C282Y/C282Y mutation were more common in the West and North regions of the state. Further studies are needed to investigate the association of these cases with environmental risk factors such as lifestyle and eating habits, including the consumption of iron-fortified foods. It is also suggested that further studies should confirm whether additional pathological conditions (such as ferroportin disease and metabolic syndrome that can be associated with iron overload) exist in cases of non-C282Y/C282Y *HFE*-hemochromatosis, as well as the presence of other mutations involved in iron regulation, in addition to the *HFE* gene. 
